# 50 YEARS OF NEWBORN SCREENING FOR CONGENITAL HYPOTHYROIDISM: EVOLUTION OF INSIGHTS IN ETIOLOGY, DIAGNOSIS AND MANAGEMENT: Management during pregnancy and long-term outcomes of adult patients with congenital hypothyroidism

**DOI:** 10.1530/ETJ-25-0125

**Published:** 2025-07-11

**Authors:** Charles Mégier, Dominique Luton, Athanasia Stoupa

**Affiliations:** ^1^ Gynecology and Obstetrics Department, Bicêtre Hospital, AP-HP, Paris Saclay University, Le Kremlin-Bicêtre. France; ^2^ Paris Regional Newborn Screening Program Department, Pediatric Endocrinology, Diabetology and Gynecology Department, Necker Children’s University Hospital, Paris Cité University, ENDO-ERN (European Reference Network) for Rare Endocrine Diseases, Assistance Publique Hôpitaux de Paris, Paris, France

**Keywords:** congenital hypothyroidism, pregnancy, long-term management

## Abstract

Congenital hypothyroidism (CH) is a lifelong condition, diagnosed shortly after birth through newborn screening in one-third of countries worldwide. When diagnosed and treated early in nonsyndromic CH, most patients exhibit similar fertility, metabolic and cardiovascular health, bone health, and quality of life compared to unaffected individuals. Special precautions are required for adult female patients with CH during pregnancy to ensure optimal management and to prevent serious maternal and fetal complications. In this review, we summarize the current knowledge on comorbidities and the long-term management of adults with CH, with a particular focus on pregnancy.

## Introduction

Congenital hypothyroidism (CH) is the most common neonatal endocrine disorder and a leading cause of preventable intellectual disability when not diagnosed and treated promptly. The introduction and widespread implementation of newborn screening programs for CH over the past 50 years in most industrialized countries have dramatically improved health and neurodevelopmental outcomes (1). Optimal long-term management of CH, particularly during the transition from pediatric to adult care, remains a significant challenge. Effective adult care requires a comprehensive approach to ensure the best possible health outcomes. Health care professionals must ensure continuity of care and address the specific needs of adult patients, including issues related to pregnancy and reproductive health. In this review, we present current knowledge and highlight the challenges associated with the long-term management and outcomes of adult patients with CH, with a particular focus on pregnancy.

## Thyroxin: a key player in materno-fetal thyroid metabolism

**Figure 1:**
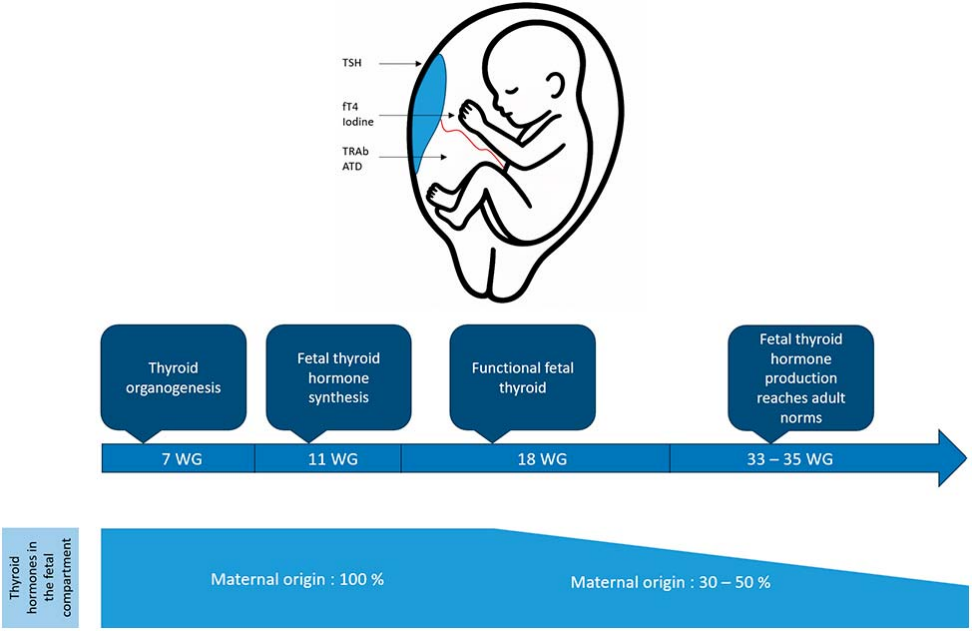
Thyroid metabolites transplacental passage and main steps of fetal thyroid development. TSH, thyroid-stimulating hormone; fT4, free T4; TRAb, thyroid receptor antibodies; ATD, antithyroid drug.

Thyroid organogenesis is initiated at day 24 of human gestation and is mostly finalized by the end of the first trimester of gestation, around 12 weeks of gestation (WG) (2). Fetal thyroid hormone (TH) synthesis begins around 10 WG (Fig. 1), and fetal free thyroxine or T4 (fT4) can be detected in the fetal circulation by 11 WG (3). However, it is not entirely functional before 17–19 WG. Until then, maternal TH are mandatory for fetal physiological development, and the fetus relies entirely on them in the first trimester (4). Maternal T4 crosses the placenta via a large variety of specific transporters such as LAT1, LAT2, and MCT8 (5). However, the placental barrier prevents maternal free triiodothyronine or T3 (fT3) from passing through. After the beginning of the second trimester, TH from both maternal and fetal thyroid glands are present in the fetal circulation. Fetal TH synthesis is fully dependent on maternal iodine transport through the placenta via the sodium/iodide symporter (NIS) or via pendrin (6). The fetal concentration of total and free circulating T4 increases throughout pregnancy and reaches the mean adult values at approximately 36 WG. This reflects the increased maturation of the fetal pituitary gland and liver.

## Fecundity and management during pregnancy

Reproductive function in individuals with isolated CH develops normally when the condition is adequately treated from childhood. When thyroid function is well controlled, the need for assisted medical procreation is not significantly higher than in the general population, and no significant delay in conception is observed (7, 8). However, poorly controlled thyroid function may lead to higher rates of infertility (9).

Maternal overt hypothyroidism during pregnancy has been associated with miscarriage, gestational hypertensive disorders, and postpartum hemorrhage (5, 10, 11, 12). A precise balance of TH in the fetal compartment is essential to ensure normal embryogenesis, brain development, and fetal growth. For example, neuronal proliferation, migration, axonal growth, dendritic synaptogenesis, neural differentiation, and myelination all require adequate TH levels. Fetal hypothyroidism is associated with adverse neurological outcomes (4). Children born to mothers with inadequately controlled hypothyroidism are at higher risk of lower intellectual quotient IQ, neurodevelopmental disorders, and a higher incidence of attention-deficit/hyperactivity disorder (ADHD) compared to those born to euthyroid mothers (13, 14). Neurodevelopmental prognosis is closely linked to maternal free T4 levels. Recent data also highlight the impact of both maternal hypothyroxinemia and hyperthyroxinemia on lower infant IQ and reduced cerebral gray matter volume (13, 15, 16, 17). These findings advocate for precise regulation of thyroxine levels, ideally targeting median values within population-specific reference ranges.

Levothyroxine treatment (fT4) should be optimized in women with CH who are planning a pregnancy (18). Before conception, target TSH levels range from the lower reference limit to 2.5 mIU/L (19, 20). Women already receiving fT4 should increase their dose by 20–50% during the first trimester, either systematically or gradually. Treatment should be monitored after 4 weeks and adjusted according to TSH levels. The target TSH level should be below 2.5 mIU/L during the first trimester and below 3.0 and 4.0 mIU/L during the second and third trimesters, respectively. Free T4 (fT4) should be maintained within the normal range. Dose adjustments should be based on fT4 levels, as hypothyroxinemia is a known risk factor for impaired neurological development, particularly before 14 weeks of gestation. Conversely, values approaching the upper limit of normal should be avoided.

In pregnant women with CH, levothyroxine dosage should be adjusted to maintain fT4 concentrations above the mean or median value of the trimester-specific reference interval. Pharmacologic preparations containing L-triiodothyronine (free fT3 or fT3), such as Cytomel® or liothyronine, should be discontinued before pregnancy. This is because exogenous fT3 can suppress maternal fT4 levels, thereby reducing the amount of fT4 available to the fetus, as fT4 is the only form that crosses the placenta in significant amounts.

Immediately after delivery, the fT4 dose can be reduced to the preconception level, and TSH should be reassessed 6 weeks postpartum. Regardless of fT4 supplementation, all pregnant women should ensure an iodine intake of 250 μg per day (19, 20, 21).

## Screening for congenital hypothyroidism in descendants

CH can be divided into primary (the most frequent form) and central. Primary CH is due to: i) dyshormonogenesis, encompassing various forms of enzymatic failure involved in the pathway of thyroid hormone synthesis and secretion in a normally located thyroid; ii) thyroid development disorders called thyroid dysgenesis (encompassing athyreosis, thyroid ectopy, hemiagenesis, and hypoplasia) (22, 23). CH can be isolated or syndromic, permanent or transitory.

### Dyshormonogenesis

Genetic defects concern genes encoding for components of the thyroid hormone biosynthesis machinery (*SLC5A5/NIS*, *TG*, *TPO*, *SLC26A4/PDS*, *SLC26A7*, *IYD*, *DUOX2/DUOXA2*) (23, 24). Patients with dyshormonogenesis are at high risk for developing thyroid nodules during adolescence and adulthood, underlining the necessity to undergo regular thyroid ultrasounds (25). In most cases, dyshormonogenesis is an autosomal recessive disease with a recurrence risk of 25%. In such instances, ongoing pregnancies should be screened as early as 22 WG to detect fetal goiter, allowing for timely *in utero* treatment. This can be achieved through intra-amniotic fT4 injections or by administering prodrugs, thus preventing mechanical complications of the goiter, such as polyhydramnios and premature delivery (26). We have shown that the primary effect of the treatment reduces thyroid size rather than ensuring optimal fT4 levels in the fetal bloodstream, at least on the cord blood sampling at delivery (27). Meanwhile, maintaining correct maternal fT4 levels should protect the fetus from neurological damage.

With early neonatal screening and appropriate treatment, the prognosis for these fetuses should not differ significantly from that of non-dyshormonogenetic CH. In all cases, specific follow-up should be arranged with a pediatric endocrinologist, including appropriate genetic screening and counseling.

### Thyroid dysgenesis

Currently, due to the diverse presentations and the wide range of implicated genes, screening for CH relies primarily on early neonatal assessment of TSH and/or T4 levels, typically performed on a dried blood spot (‘Guthrie card’) between day 2 and day 3 of life, often in combination with other metabolic or genetic screenings (28). A molecular defect is identified in only 5–10% of patients with TD, with a complex mode of inheritance (22). However, special attention should be paid to associated malformations in syndromic cases of TD, as in the case of *NKX2-1*, *PAX8*, *FOXE1,* and *NKX2-5* mutations.

Concerning pregnancy in women affected by CH, maintaining adequate maternal fT4 levels will protect the fetus from neurological damage. Furthermore, as long as early neonatal screening is conducted and appropriate treatment is initiated, no significant developmental impairments are expected.

In the future, specific fetal thyroid screening – such as evaluating for athyreosis or hypoplasia – could be explored primarily for research purposes. However, its clinical benefit is currently considered limited.

### Thyroid failure included in a syndromic feature

In theory, CH associated with other syndromic disorders (e.g. Pendred syndrome, *FOXE1*, *PAX8*, *NKX2*-*1*, *GLIS3*, *JAG1*, *NKX2*-*5*, and *GNAS*) could allow for prenatal screening to identify recurrence. However, alternative screening methods, primarily genetic testing or evaluation of associated malformations, may also be utilized. Whether such screening is justified remains a subject of debate and probably depends on the associated syndrome.

## Cognitive and educational outcomes

Before newborn screening programs for CH, untreated CH led to severe intellectual disability (29, 30, 31). Introduction of NBS programs for CH has significantly improved prognosis for detected children. However, approximately 70% of newborns worldwide still lack access to NBS for CH, predominantly in African and Asian countries, accounting for a significant part of annual births (28). Early-treated CH patients have a mean IQ 20 points higher than those diagnosed late (32). Nevertheless, despite screening and early treatment, some patients still experience subtle deficits in memory, attention, language, and motor skills (22, 33, 34, 35, 36). Concerning educational achievements, some studies have reported low levels in school attainment and progression through different grades, but this fact was associated with the severity of CH and the inadequacy of the therapeutic regimen (37, 38). Affected individuals may require additional educational support and long-term cognitive monitoring.

## Behavioral and psychological impact

Most adult CH patients lead normal lives, but some struggle with emotional and behavioral issues (38, 39). Higher rates of anxiety and depressive moods have been reported for some patients, while a recent nationwide study in Finland did not support evidence for severe psychiatric comorbidity in CH adult patients (39, 40). Furthermore, many CH young adults continue living with parents, possibly indicating delayed independence. Further large studies are needed to study their behavioral development during adulthood.

## Quality of life and social integration

Most patients successfully integrate into society, but some may experience minor disadvantages in education and employment (36, 38, 39, 41). Young adults with CH tend to have slightly lower self-esteem and concerns about mental performance.

In summary, adult patients with CH may face behavioral and psychological challenges, including subtle health impairments, reduced socioeconomic attainment, and neuropsychological deficits (42). These outcomes are influenced by factors such as treatment adequacy, socioeconomic status, severity of the disease, presence of other chronic diseases, and potential thyroid hormone hyposensitivity (33, 38, 42). Continuous monitoring and tailored interventions are essential to enhance the quality of life and psychological well-being of individuals with CH.

## Hearing and neurosensory development

TH play a crucial role in the development and function of the cochlea and hearing mechanisms (43, 44). Their influence extends from embryonic development to postnatal maturation, ensuring proper auditory function. In cases of TH deficiency, as in cases of CH or iodine deficiency (45), hearing impairment concerns delayed cochlear maturation, incomplete differentiation of hair cells and supporting cells, defective synapse formation in the auditory nerve, and sensorineural hearing loss. Even with early diagnosis and treatment, CH patients are three to four times more likely to develop hearing impairment (9.5%) than the general population (2.5%) (46, 47, 48). Hearing loss was shown to be associated with the type of CH (more common in athyreosis) and the severity of CH cases (47). Most affected individuals experience bilateral, mild-to-moderate sensorineural hearing loss, particularly at high frequencies. Hearing defects can also be part of the clinical phenotype in several syndromic CH cases or rare thyroid hormone receptor disorders (49, 50, 51, 52, 53). Given the crucial role of TH ranging from development and morphogenesis to the fine-tuning of the auditory process, regular audiological evaluations throughout childhood and early adulthood are recommended in patients with CH (18).

## Metabolic and cardiovascular health

CH, if not properly managed, can impact metabolic and cardiometabolic health in young adults. Studies have shown that with early and appropriate fT4 treatment, patients with CH generally maintain a normal body mass index (BMI) and body composition comparable to the general population (54, 55). However, they may experience an earlier adiposity rebound and a higher likelihood of being overweight or obese, affecting up to 37% of young adults with CH. Cardiovascular evaluations in young adults with CH have shown normal blood pressure, glucose levels, lipid metabolism, and carotid intima-media thickness (IMT) (56). However, inconsistent treatment adherence may contribute to subtle cardiovascular impairments, such as reduced exercise capacity, diastolic dysfunction, increased IMT, and mild endothelial dysfunction (57). While the long-term significance of these changes is not yet fully understood, they emphasize the need for strict treatment adherence to support optimal metabolic and cardiovascular health. In addition, long-term fT4 therapy in young adults with CH may be linked to impaired diastolic function, reduced exercise capacity, and increased IMT (57). These potential cardiovascular effects reinforce the necessity of regular monitoring and personalized treatment approaches to prevent adverse outcomes. In conclusion, while early and adequate management of CH helps maintain favorable metabolic and cardiometabolic health, ongoing monitoring and lifestyle modifications are essential to address potential health challenges associated with the condition. Special attention should also be paid to congenital heart defects associated with primary CH; this could be in a known genetic background, as in *NKX2-5* mutations, or other rare cases of syndromic CH. Reported frequency of heart defects in CH varies between 3–11% (22, 48, 57, 58, 59), contrary to 0.5–0.8% in all live births. In all cases, specific clinical care and follow-up at adulthood are required for these patients.

## Bone health

TH have a crucial role in skeletal growth and bone homeostasis (60). Newborns and infants with CH have delayed bone maturation, progressively restored under fT4 substitutive treatment. Long-term research on children and young adults with CH has indicated normal bone mineral density, implying that early initiation and appropriate fT4 treatment do not negatively impact bone health (61, 62, 63). Special caution should be taken for excessive fT4 treatment, which may accelerate bone turnover, leading to greater bone resorption than formation, and ultimately causing progressive bone loss (64, 65).

## Conclusions

To conclude, almost 50 years after the introduction of newborn screening for CH, most diagnosed patients have reached adulthood, underlining the importance of enhanced awareness of the disease and the associated comorbidities. The management of CH in adulthood necessitates a comprehensive and multidisciplinary approach to optimize long-term health outcomes. Continuity of care is essential, particularly during the transition from pediatric to adult services. Finally, healthcare providers must address the specific clinical considerations associated with adult life, including reproductive health, fertility, and the management of thyroid hormone requirements during pregnancy.

## Declaration of interest

The authors declare that there is no conflict of interest that could be perceived as prejudicing the impartiality of the work reported.

## Funding

This research did not receive any specific grant from any funding agency in the public, commercial, or not-for-profit sector.
